# *Mycobacterium microti* Infection in Wild Boar (*Sus scrofa*): Histopathology Analysis Suggests Containment of the Infection

**DOI:** 10.3389/fvets.2021.734919

**Published:** 2021-09-13

**Authors:** Claudio Pigoli, Vito Tranquillo, Lucia Rita Gibelli, Alessandra Gaffuri, Giovanni Loris Alborali, Maria Pacciarini, Mariagrazia Zanoni, Maria Beatrice Boniotti, Giuseppe Sironi, Mario Caniatti, Valeria Grieco

**Affiliations:** ^1^Laboratorio di Istologia, Sede Territoriale di Milano, Dipartimento Area Territoriale Lombardia, Istituto Zooprofilattico Sperimentale della Lombardia e dell'Emilia-Romagna, Milan, Italy; ^2^Department of Veterinary Medicine (DIMEVET), University of Milan, Lodi, Italy; ^3^Sede Territoriale di Bergamo, Dipartimento Area Territoriale Lombardia, Istituto Zooprofilattico Sperimentale della Lombardia e dell'Emilia-Romagna, Bergamo, Italy; ^4^Sede Territoriale di Brescia, Dipartimento Area Territoriale Lombardia, Istituto Zooprofilattico Sperimentale della Lombardia e dell'Emilia-Romagna, Brescia, Italy; ^5^Dipartimento Tutela e Salute Animale, Centro di Referenza Nazionale per la Tubercolosi da Mycobacterium bovis, Istituto Zooprofilattico Sperimentale della Lombardia e dell'Emilia-Romagna, Brescia, Italy

**Keywords:** granuloma, histopathology, *Mycobacterium microti*, wild boar, animal tuberculosis

## Abstract

The European wild boar (WB) (*Sus scrofa*) population has rapidly expanded over the years, raising public health concerns over the species reservoir of several pathogens, including *Mycobacterium microti* (*Mm*), a *Mycobacterium tuberculosis* complex member. In this study, we aimed to investigate the *Mm* natural infection in WB in Lombardy and Emilia Romagna Italian regions by statistically evaluating the granulomatous lesions' histological features and *Mm* microbiological isolation. We analyzed 103 WB retropharyngeal and submandibular lymph nodes (LNs) for *Mm* identified by *gyrB* PCR-restriction fragment length polymorphism, and were retrospectively selected and histologically assessed. For each sample, Hematoxylin-eosin and Ziehl–Neelsen stained slides were evaluated. Considered histological variables were: the number of granulomas, size and maturational stage of granulomas, granulomas completeness within the section, number of multinucleated giant macrophages (MGMs), and acid-fast (AF) bacilli per granuloma. Furthermore, *Mm* microbiological results were also considered. *Mm* microbiological isolation was negatively influenced by granulomas maturation and positively affected by AF bacilli's presence within the section. Granuloma maturation was positively influenced by granuloma size and granuloma incompleteness and negatively affected by the number of granulomas in the section and the number of MGMs within the granuloma. The results indicate that granuloma maturation should ensures an efficient containment of *Mm* infection in the WB, suggesting that the intra-species transmission of the disease might be an unlikely event.

## Introduction

The European wild boar (WB) (*Sus scrofa*) population has rapidly expanded over the last decades (1, 2). This dramatic increase has been attributed to multiple factors, including more favorable climatic conditions, a reduction in natural predators, and, in particular, a decrease in human hunting activity, which was the primary source of WB population control (3). Even if WB numerical and geographic expansion induced a positive boost to its meat market, it also increased contact among humans, domesticated animals, and WBs, raising public health concerns (4, 5).

WB is considered a reservoir of several pathogens and parasites that can be transmitted to humans and animals (2, 4). Several species belonging to the *Mycobacterium tuberculosis* complex (MTBC) have been identified in wild animals, and for this reason, several countries engage in wildlife monitoring and management. Specifically, *M. bovis, M. caprae*, and *M. microti* (*Mm*) are the MTBC members identified in WB, and the role of the latter as a reservoir for these pathogens is demonstrated or highly suspected in various ecosystems (1, 6–10). In particular, WB tuberculosis seems to have hindered eradicating the disease in different zootechnical–rural realities, endangering animal products' consumption, hampering animal movement toward tuberculosis free areas, and burdening the costs associated with veterinary controls (2).

*Mm* is an MTBC member first described in 1937 in the field vole (*Microtus agrestis*)—the adapted host (1, 11). Hardly differentiable from other MTBC members applying phenotypic assays, *Mm* is also characterized by an extremely challenging and slow *in vitro* growth. These features had probably led to an underestimation of *Mm* presence in the past when specific molecular tests were unavailable (1). *Mm* lacks the RD1^mic^ genomic region, a region that partially overlaps the RD1 deletion responsible for the attenuation of *M. bovis* bacillus Calmette–Guérin vaccine strain (12). Due to this deletion, *Mm* has probably relatively low pathogenicity for humans, which is why non-attenuated strains of *Mm* have been used as a vaccine against human tuberculosis; however, *Mm*-induced disease has been increasingly reported over the years (11, 12). *Mm* has been isolated from cattle, goat, domestic pig, South American camelids, cat, dog, wild and pet ferrets, WB, badger, meerkat, wood mice, and humans (7, 13–16). In particular, *Mm* has been isolated from both immunocompetent and immunocompromised human patients presenting with pneumonia and, occasionally, encephalitis and peritonitis (11, 12, 16, 17).

In Northern Italy, the prevalence of *Mm* in WB has been reported to be between 3.26 and 7%, depending on geographical area and abundance of the WB population (1). Even if the influence of animal abundance suggests an intra-species pathogen transmission, the lack of data regarding population and infection prevalence in small rodents hampers certainty of epidemiological dynamics of the infection (1, 7). In WB, *Mm*-induced lesions are restricted to retropharyngeal and submandibular lymph nodes (LNs), and generalized diseases are not reported (1, 7). Induced lesions are consistent with classical tubercular granulomas and usually contain scant acid-fast (AF) bacilli (2).

In animals, tubercular granulomas can be histologically classified into maturational stages based on cellular composition, degree of capsulation, presence, and features of central necrosis, and degree of mineralization. In particular, the classification system described by Wangoo et al. in 2005 is used to classify animals' LN tubercular lesions (18). This classification, including four maturational stages, was initially designed to study *M. bovis*-induced LN lesions in experimentally infected cattle. It was later applied to investigate lesions induced by MTBC members in the LNs of animals other than cattle, such as those caused by *M. bovis* and *M. caprae* in naturally infected WB (10). Based on histological features, identified stages imply specific inflammatory-biochemical patterns that drive granuloma development from initial simple inflammatory aggregates to capsulated and mineralized necrotic lesions (18). Since the defined stages represent the standard maturative steps of granulomas, an eventual alteration of this evolutive path suggests an imbalance in host–pathogen interaction, with altered pathogen containment. Thus, this classification allows us to study the pathogenesis of animal tuberculosis and can help to explain some of the epidemiological features of such diseases (10). Furthermore, other histological features, such as a high number of multinucleated giant macrophages (MGMs), phagocytic cells deriving from activated macrophages fusion, and a high AF bacilli load, are associated with low mycobacteria containment (10, 19, 20). Consequently, animals harboring granulomas with histological features consistent with poor mycobacteria containment are expected to be more prone to contaminate the environment and transmit the infection than animals whose granulomas suggest an efficient pathogen containment (21).

This study was aimed to investigate histologically the lesions induced by *Mm* in the LNs of naturally infected WBs, evaluating the possible effect of the considered histological parameters on the granuloma maturational stage and the cultural isolation of *Mm* from the LNs.

## Materials and Methods

### Sample Retrospective Selection

From the electronic archives of Istituto Zooprofilattico Sperimentale della Lombardia e dell'Emilia Romagna (IZSLER), 120 WB retropharyngeal and submandibular LNs, sampled according to Boniotti et al. (2) and conferred in the years 2014–2018, were retrieved. These samples were collected according to the Lombardy and Emilia Romagna regions wildlife monitoring and control plan, which involves post-mortem inspection of all hunted WBs (22).

Briefly, LNs showing lesions compatible with tuberculosis were divided into two parts: one part underwent molecular analysis and microbiological isolation. The other part was formalin-fixed and routinely processed for histology. Molecular analysis was performed directly on the fresh tissue sample and consisted in a *gyrB* PCR-restriction fragment length polymorphism analysis. Microbiological isolation was performed after decontamination onto Löwenstein-Jensen and Stonebrink solid media with addition of pyruvate, and into modified Middlebrook 7H9 broth incubated in a Bactec MGIT 960 system (Becton, Dickinson and Company, Sparks, MD). Incubation was carried out at 37°C for 18 weeks, and the isolates were identified as *Mm* using a *gyrB* PCR-restriction fragment length polymorphism analysis. Considering histology, after paraffin-embedding, 4 μm thick sections were cut from the paraffin block and were dewaxed, rehydrated, and Hematoxylin-Eosin (HE) and Ziehl-Neelsen (ZN) stained (2).

In this study, the 120 WB LNs retrieved were both positive for *Mm* by *gyrB* PCR-restriction fragment length polymorphism applied directly on fresh tissue, and for the presence of at least a granuloma in HE stained histological sections (1, 2).

After a preliminary view of the HE stained slides, autolytic samples were excluded. For each enrolled case, the results of *Mm* microbiological isolation were also recorded.

### Histopathology

For each case, two 4 μm thick serial sections were cut from the formalin-fixed and paraffin-embedded block and subjected to HE and ZN staining. HE stained slides were then scanned at 20× magnification using the precise mode of the VISIA NaviFIVE bright field slide scanner with automatic focus detection.

Physical HE stained slides were then carefully viewed at 40× magnification using an OLYMPUS CX31 bright field microscope equipped with coplanar objectives to identify granulomatous lesions. Using Visa VISIA Navi Viewer software, each lesion was also identified in the scanned slide, and its area was calculated. Virtual HE stained slides were also used to associate the study parameters (area, granuloma stage, number of MGMs, and the number of AF bacilli) to each lesion. We also recorded if the lesions were entirely or incompletely depicted in the section.

**Figure 1:**
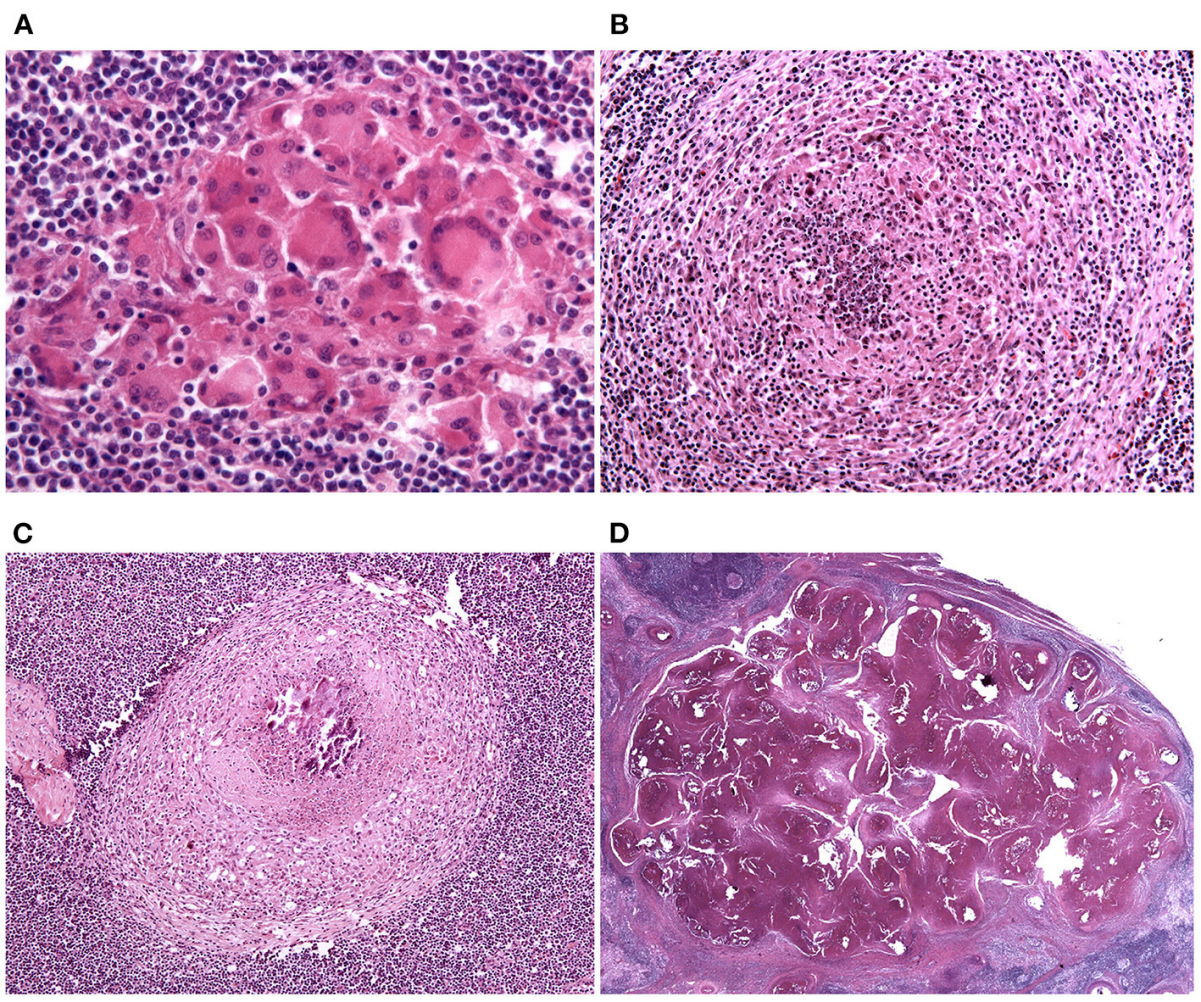
Lymph node, granuloma maturational stages. **(A)** Stage 1 (initial) granulomas are composed of an unencapsulated cluster of epithelioid macrophages. HE stain, 40×. **(B)** Stage 2 (solid) granulomas are enclosed partly or entirely by a thin capsule and a minimal necrotic core, composed of necrotic inflammatory cells, is sometimes visible. HE stain, 20×. **(C)** Stage 3 (minimal necrosis) granulomas are fully encapsulated and a central necrotic core, composed of a variably mineralized caseous material, is present. HE stain, 10×. **(D)** Stage 4 (necrosis and mineralization) granulomas are multicentric, thickly encapsulated, with abundant and extensively mineralized caseous necrosis. HE stain, 0.80×.

Granulomas were classified into four stages, according to Wangoo et al. (18). Stage 1 (initial; Figure 1A) granulomas are composed of an unencapsulated cluster of epithelioid macrophages (polygonal to elongated mononucleated phagocytes, usually larger than activated macrophages, and characterized by abundant cytoplasm) with interspersed lymphocytes, neutrophils and MGMs. Stage 2 (solid; Figure 1B) granulomas are still composed of epithelioid macrophages, lymphocytes, neutrophils and MGMs but are enclosed partly or entirely by a thin capsule; a minimal necrotic core, consisting of necrotic inflammatory cells, is sometimes visible. Stage 3 (minimal necrosis; Figure 1C) granulomas are fully encapsulated and have a central necrotic core composed of a variably mineralized caseous material; the necrotic core is lined by epithelioid macrophages admixed with MGMs and, in the outermost parts, lymphocytes clusters and occasional neutrophils are present. Stage 4 (necrosis and mineralization; Figure 1D) granulomas are multicentric, thickly encapsulated, with abundant and extensively mineralized caseous necrosis surrounded by epithelioid macrophages, MGMs and, in the outermost parts, lymphocytes clusters.

The number of MGMs per lesion was counted in physical HE stained slides at 40× magnification.

Each lesion was then found in the corresponding ZN-stained section and accurately and wholly viewed at 100× magnification with oil immersion to count AF bacteria.

### Statistics Analysis

For the statistical data elaboration, two outcome variables of interest were identified:

1. The microbiological status of the LNs (dichotomous variable: Positive/Negative);
2. The histological stage of granulomas (ordinal variable with four levels: stages 1, 2, 3, and 4).

Based on the outcome variable's nature, we adapted specific Bayesian regression models to identify which variables among those measured significantly influenced the outcomes (23).

#### The Microbiological Status of the LNs

In the applied model, the statistical unit was the single LN, and predictors used were:

a) AF bacilli: the presence of AF bacteria in LN granulomas. This variable was obtained by calculating, for every LN, the average number of AF bacteria present in the various granulomas and then categorizing the LN itself as AF = 1 (i.e., the presence at least of one AF bacteria) when the average was >0, and AF = 0 (i.e., the absence of AF bacteria) in the case of averages equal to 0;
b) MGM: the presence of MGMs in the LN granulomas. The categorization of LNs based on the presence of MGMs in the granulomas was obtained following a method similar to the method performed for AF bacilli. LNs were categorized as MGM = 1 when at least one MGM has been counted in one of its granulomas. On the contrary, an LN was classified as MGM = 0 when no MGMs were observed in its granulomas.
c) G1, G2, G3, G4: to understand the influence of the granulomas histological stage on the microbiological status of LNs, we constructed four new variables identifying the proportions of granulomas of different stages in each LN. For each LN, we calculated the number of granulomas of the different histological stages. Then, we divided it by the total number of granulomas per LN, thus obtaining a value between 0 and 1, which corresponds to the proportion of granulomas of different stages. Precisely, G1, G2, G3, and G4 indicate the proportion of granulomas of stages 1, 2, 3, and 4 concerning the total number of granulomas present in an LN.

The microbiological status (i.e., *Mm* microbiological isolation) outcome variable was modeled using a Bayesian General Linear Model (24). The observed positive microbiological status (M) was considered as Bernoulli realizations of a random process with probability p to be positive:


$$M_{i}\sim Bernoulli(p)$$

Then p was modeled using a logit link function:


logit(p) = α+βX

where X is the predictor's design matrix, α is the intercept parameter, and β is the matrix of predictor's regression coefficients. For both parameters (α and β), weakly uninformative priors were selected according to McElreath (24):


$$\begin{array}{l} \alpha \sim Normal(0,\;1)\\ \beta \sim Normal(0,\;1) \end{array}$$

The model parameter estimates were obtained by sampling with the Hamiltonian Monte Carlo algorithm. For this model, the brms package was used as the interface of the STAN implemented in R in the rstan package (25–27). Bayesian posterior estimates of the parameters were then summarized in a table using the median of the posterior distribution, the 95% credibility intervals, the Probability Direction (PD), as a measure of the importance of the effects of the parameters, and the Region of Practical Equivalence (ROPE) as an index of significance as reported by Makowski et al. (28).

#### The Histological Stage of Granulomas

In the applied model, the statistical unit was the single granuloma, and predictors used were:

a) lnGrArea: log of the granuloma area;
b) laf: log of the number of AF bacteria per granuloma;
c) lmgm: log of the number of MGMs per granuloma;
d) micro: microbiological state of the LNs (Positive/Negative);
e) Grcompl: status of complete/incomplete granuloma;
f) lngr: log of the number of granulomas per LN;
g) Idlymph: code identifier of LN.

In our case, a Bayesian ordinal regression model was fitted according to Bürkner and Vuorre (29). In our study, a Sequential Model was used because the assumption of the model concerning the variable outcome is consistent with the histological development of the granulomas (the histological stage is the expression of a sequential process for which, for example, a granuloma is classified as stage 4, after having “evolved” from the previous stages). In our case, the Sequential Model was further complicated by the data's hierarchical structure, whereby observations made at the granuloma level were nested under the LNs. So a multilevel ordinal sequential model was fitted, with Idlymph as a random effect.

In the Sequential Model the dependent variable Y results from a counting process and is truly ordinal in the sense that in order to achieve a category *k*, one has to first achieve all lower categories 1 to *k* − 1. For every category *k* ∈ {1, …, *K*} there is a latent continuos variable ${\hat{Y}}_{k}$ determining the transition between the *k* + 1*th category*. We assume that ${\hat{Y}}_{k}$ depends on the predictor term η and error ϵ_*k*_:


$$\begin{array}{l} \hat{Y_{k}}=\eta +\epsilon_{k} \end{array}$$

where η is the linear predictor: β*X* where β is the matrix of regression coefficients and *X* is the matrix of the values of predictors.

So, we can formulate the Bayesian model as:


$$\begin{array}{l} Histo\sim ordered-logit(\eta ,\;k)\\ \;\eta =\alpha_{\left[Idlinf\right]}+\beta X \end{array}$$

with these priors:


$$\begin{array}{l} k\sim Normal(0,\;5)\\ \alpha \sim Normal(\hat{\alpha },\;\sigma )\\ \hat{\alpha }\sim Normal(0,\;5)\\ \sigma \sim Student(3,\;0,\;2.5)\\ \beta \sim Normal(0,\;5) \end{array}$$

The model parameter estimates were obtained by sampling with the Hamiltonian Monte Carlo algorithm using four chains, with 4,000 iterations of which 1,000 were warmup (excluded after sampling). For this model, the brms package was used as the interface of the STAN language implemented in R in the rstan package. Bayesian posterior estimates of the parameters were then summarized in a table using the posterior distribution median, the 95% credibility intervals, the PD, and the ROPE (28).

To show how a single predictor statistically influences the distribution of the histological stage of granulomas while holding the other predictors constant, we plot the conditional effects of some relevant predictors, as described by Bürkner (25).

## Results

One hundred and twenty WB retropharyngeal and submandibular LNs, in which *Mm* was detected directly in fresh tissue samples by *gyrB* PCR-restriction fragment length polymorphism, were selected from IZSLER electronic archives. Of these, 103/120 were enrolled for histological evaluation and statistical analysis, while 17/120 were excluded due to autolysis artifacts. In 19/103 (18%), LNs *Mm* was microbiologically isolated, while in 84/103 (82%), the bacteriological examination resulted negative. Most of the LNs contained few granulomas; in some others, hundreds of granulomas were identified. Usually, when only a few granulomas were present in the section, they were classified in the highest maturational stages; conversely, when numerous granulomas were present, they were mainly of the first two maturational stages. For example, 422 is the maximum number of granulomas counted in an LN, and of them, 408, 13, and 1 were, respectively, of the first, second, and fourth maturational stage. On the contrary, in the eight LNs in which only one granuloma was detected, the latter was of the fourth maturational stage. AF bacilli were identified in 64/103 (62%) LN histological sections, while 39/103 (38%) resulted ZN negative. In the ZN positive LN sections, only one or a few granulomas usually contained AF bacilli. In such granulomas, usually <10 AF bacilli were detected. The detailed results of the analyzed variables (LN section's size, number of granulomas per LN, the size and histological stage of the granulomas, the number of MGMs, and AF bacilli per granuloma) are reported in Supplementary Table 1.

### Statistical Results

#### Influence of the Considered Variables on the LNs Microbiological Status

The statistical model results used to study how the considered variables influence the microbiological status of the LNs are shown in Table 1.

**Table 1:** Influence of the considered variables on the microbiological status of the lymph nodes (LNs).

**Parameter**	**Median**	**CI low**	**CI high**	**PD**	**ROPE percentage**
Intercept	−1.41	−3.33	0.42	0.93	0.05
G1	0.98	−0.45	2.42	0.90	0.08
G2	0.50	−1.00	2.02	0.75	0.15
G3	−1.08	−2.66	0.43	0.91	0.07
G4	−0.44	−1.92	0.98	0.72	0.16
Presence of AF bacilli	0.44	−0.56	1.45	0.81	0.20
Presence of MGMs	−0.38	−1.79	1.05	0.70	0.17

*Median, median of posterior estimated coefficient; CI low, lower limit of 95% credible interval; CI high, higher limit of 95% credible interval; PD, probability direction; ROPE Percentage, percentage of the region of practical equivalence; G1, G2, G3, G4, proportions of granulomas of different stages for each single LN; AF bacilli, acid-fast bacilli; MGMs, multinucleated giant macrophages*.

**Figure 2:**
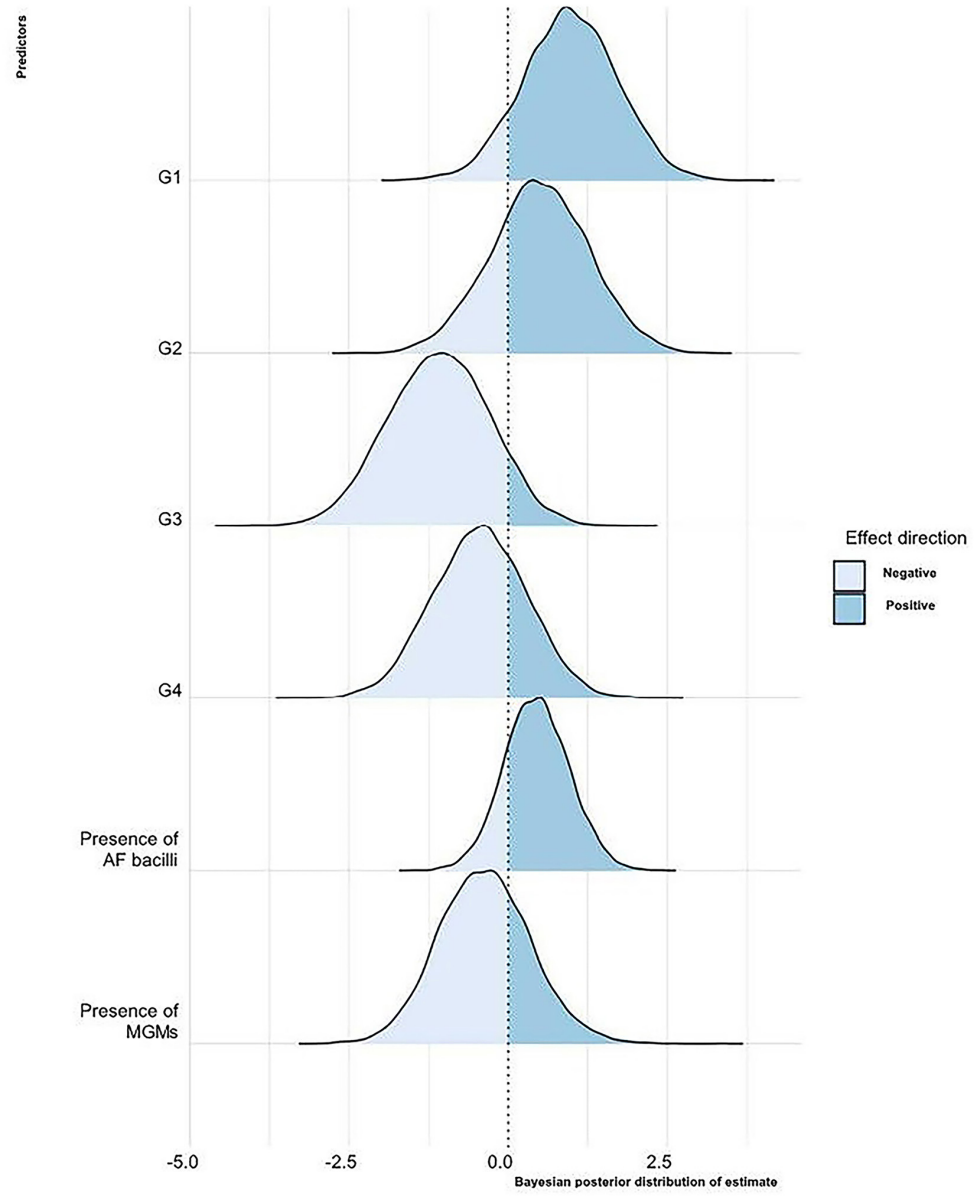
Bayesian posterior distribution of estimated coefficients of the influence of the considered variables on the LNs' microbiological status. For each predictor, the area corresponding to the values with a negative or a positive effect on the LN microbiological status is shown. G1, 2, 3, 4, proportions of granulomas of the different LN stages; AF, acid-fast; MGMs, multinucleated giant macrophages.

The Bayesian posterior distributions of estimated coefficients for each predictor with respect to the LN microbiological status are shown in Figure 2.

Briefly, keeping the value of all other variables constant, as the proportion of stage 1 granulomas per LN increases, there is a 90% probability that the LN is microbiologically positive. There is a similar effect for the variable G2, even if with a probability of 74%. As the proportion of stage 3 and 4 granulomas increases, there is a tendency to decrease the probability that the LN is microbiologically positive with a probability of 91 and 72%, respectively. In LNs where granulomas with AF bacteria are present, there is a greater probability that they are microbiologically positive in 81% of cases. On the contrary, in 70% of cases, the presence of MGMs in granulomas reduces the likelihood of the LNs to be microbiologically positive. However, the predictors' relevance in terms of distance from 0 is not particularly high, and the uncertainty of the estimates also appears to be rather large.

#### Influence of the Considered Variables on the Granulomas Histological Stage

The statistical model results used to study how the considered variables influence the histological stage of granulomas are shown in Table 2.

**Table 2:** Influence of the considered variables on granulomas histological stage.

**Parameter**	**Median**	**CI low**	**CI high**	**PD**	**ROPE percentage**
Intercept_1	−2.02	−2.69	−1.29	1.00	0.00
Intercept_2	3.84	3.16	4.60	1.00	0.00
Intercept_3	9.43	8.54	10.31	1.00	0.00
StdzArea	4.61	4.35	4.90	1.00	0.00
Completness Gr (I vs. C)	2.18	1.26	3.13	1.00	0.00
StdzLn_AF_bacilli	0.05	−0.13	0.24	0.71	0.63
StdzLn_MGMs	−0.58	−0.70	−0.45	1.00	0.00
Micro (Pos vs. Neg)	−0.11	−1.05	0.88	0.59	0.16
StdzLn_NGR	−1.50	−1.92	−1.15	1.00	0.00

*Median, median of posterior estimated coefficient; CI low, lower limit of 95% credible interval; CI high, higher limit of 95% credible interval; PD, probability direction; ROPE Percentage, percentage of the region of practical equivalence; StdzArea, standardized log of the granuloma area; Completeness Gr, completeness of the granuloma into the section; I, granuloma incompletely depicted into the section; C, granuloma completely depicted into the section; StdzLn_AF bacilli, standardized log of the number of AF bacilli per granuloma; StdzLn_MGMs, standardized log of the number of MGMs per granuloma; Micro, microbiological status of the LNs; StdzLn_NGR, standardized log of the number of granulomas per LN*.

**Figure 3:**
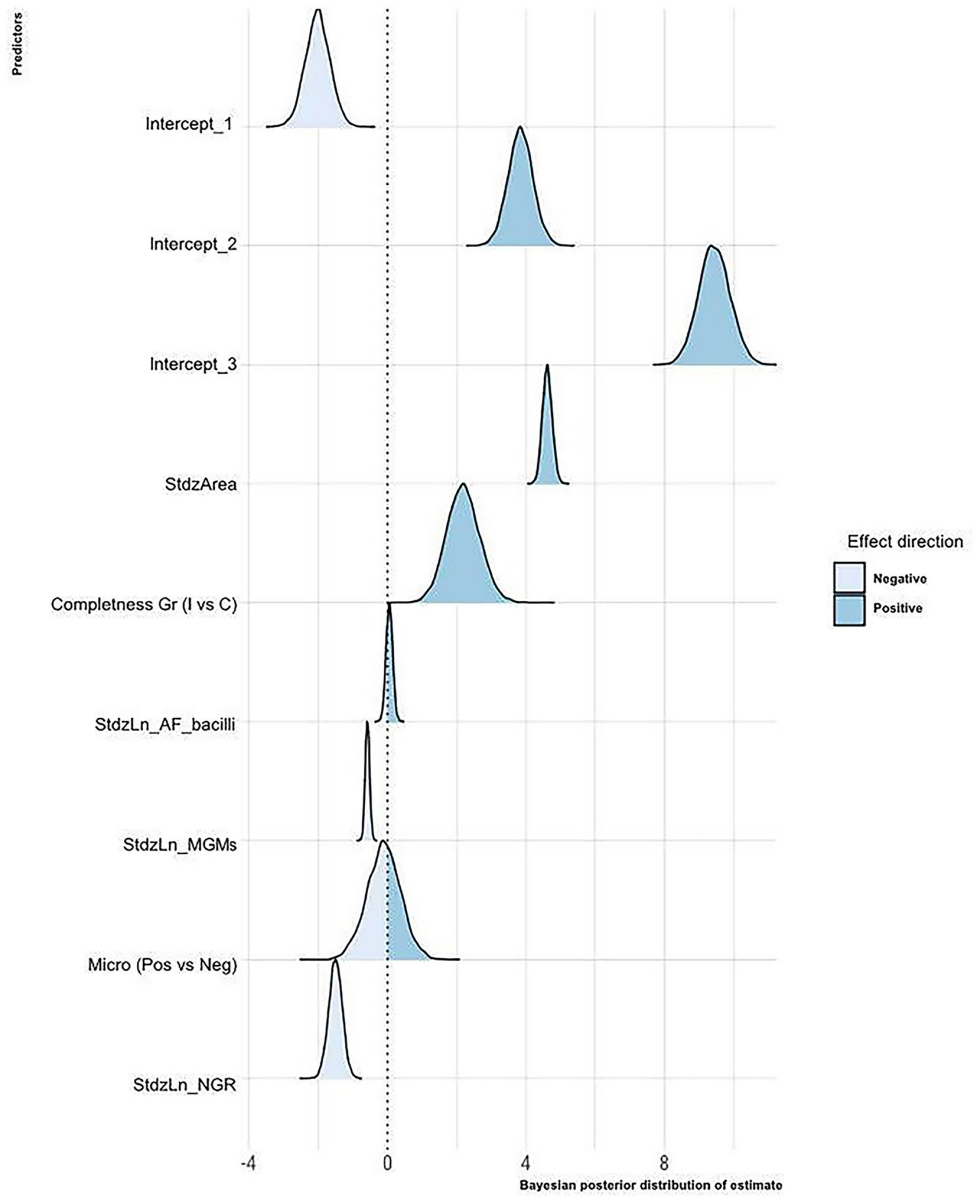
Bayesian posterior distribution of estimated coefficients of the influence of the considered variables on the granuloma histological stage. For each predictor, the area corresponding to the values with a negative or a positive effect on the granuloma histological stage is shown. StdzArea, standardized log of the granuloma area; Completeness Gr, completeness of the granuloma into the section; I, granuloma incompletely depicted into the section; C, granuloma completely depicted into the section; StdzLn_AF_bacilli, standardized log of the number of AF bacilli per granuloma; StdzLn_MGMs, standardized log of the number of MGMs per granuloma; Micro, microbiological status of the LNs; StdzLn_NGR, standardized log of the number of granulomas per LN.

The Bayesian posterior distributions of estimated coefficients for each predictor with respect to the granuloma histological stage are reported in Figure 3.

The statistical model suggests that as the granuloma's size increases, the probability that the granuloma is classified in the higher stages increases. On the contrary, as the number of granulomas in the LN increases, the probability that LN's granulomas are of high histological stages decreases. According to the model, the microbiological status does not seem relevant, whereas the probability of granulomas being classified in high stages decreases as the number of MGMs increases. Regarding the number of AF bacilli, the model suggests a positive effect of this parameter on the granuloma histological stage, but a probability of only 70% supports this result. Finally, incomplete granulomas compared to complete ones are more likely to be classified in high histological stages.

**Figure 4:**
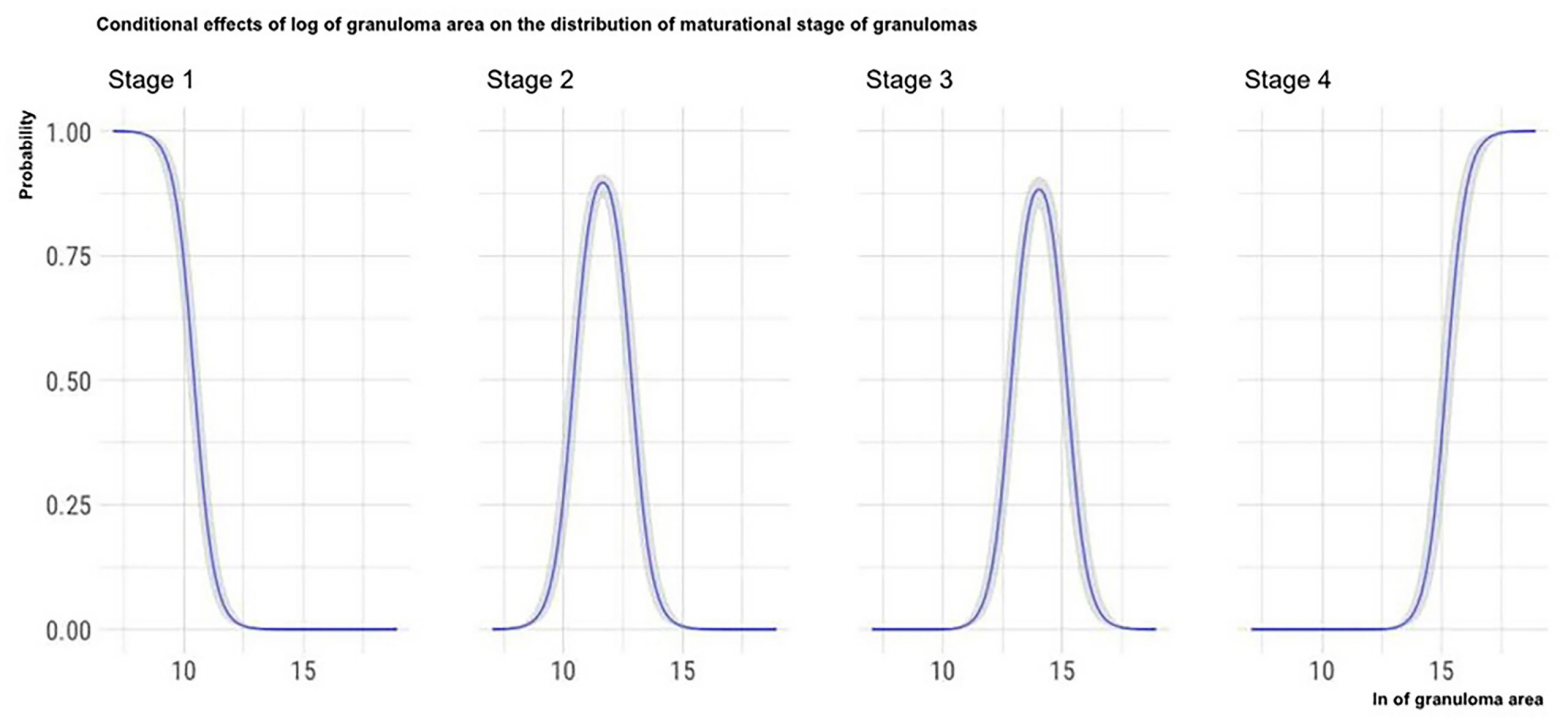
Marginal effects of granuloma size on granuloma histological stage. The figure shows that as the granulomas' area increases, the possibility that granulomas are classified in the high histological stages increases. Furthermore, the clear separation of the curves indicates that the granulomas' size can be considered a good predictor of the granulomas histological stage.

**Figure 5:**
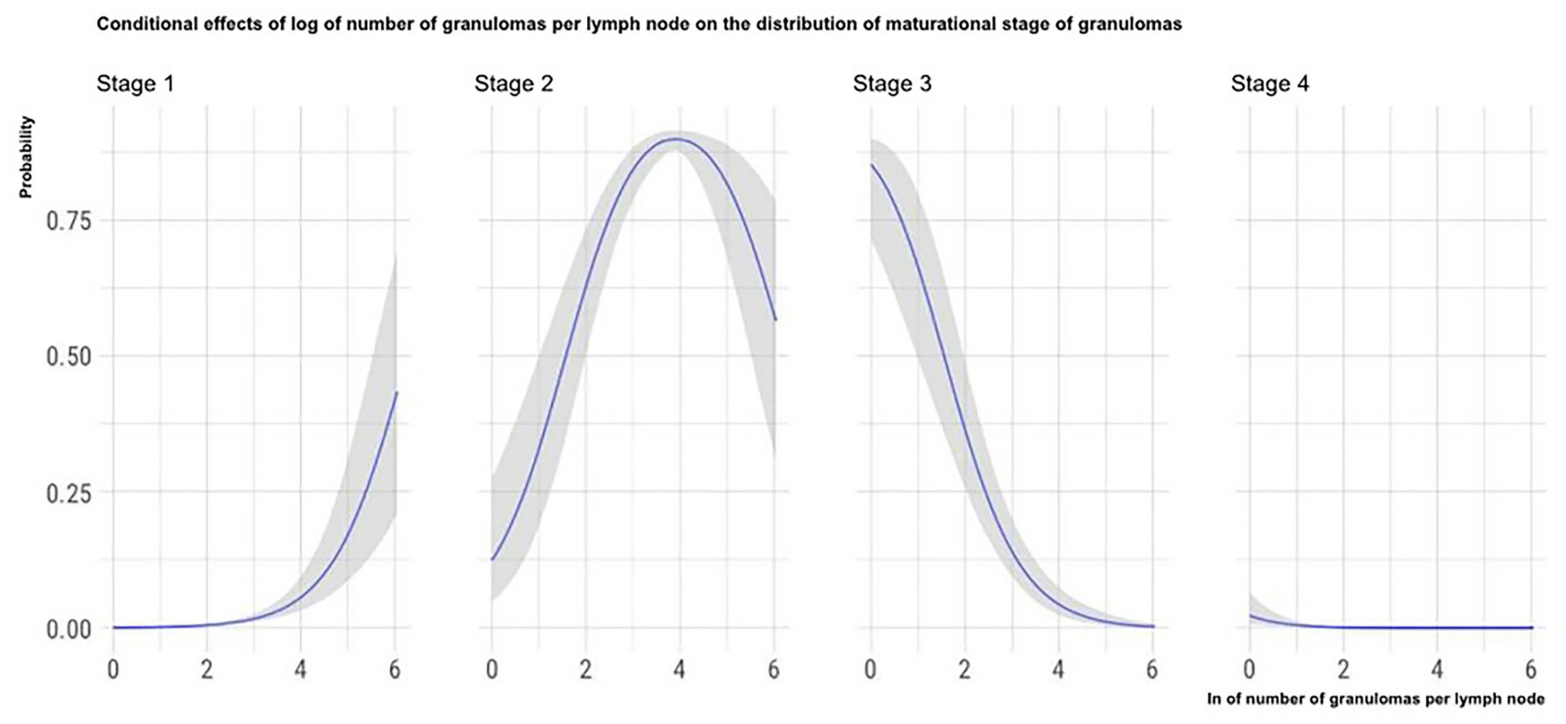
Marginal effects of the total number of granulomas per LN on granuloma histological stage. The figure shows that as the total number of granulomas per LN increases, the possibility that granulomas contained in the considered LN are classified in the high histological stages decreases.

**Figure 6:**
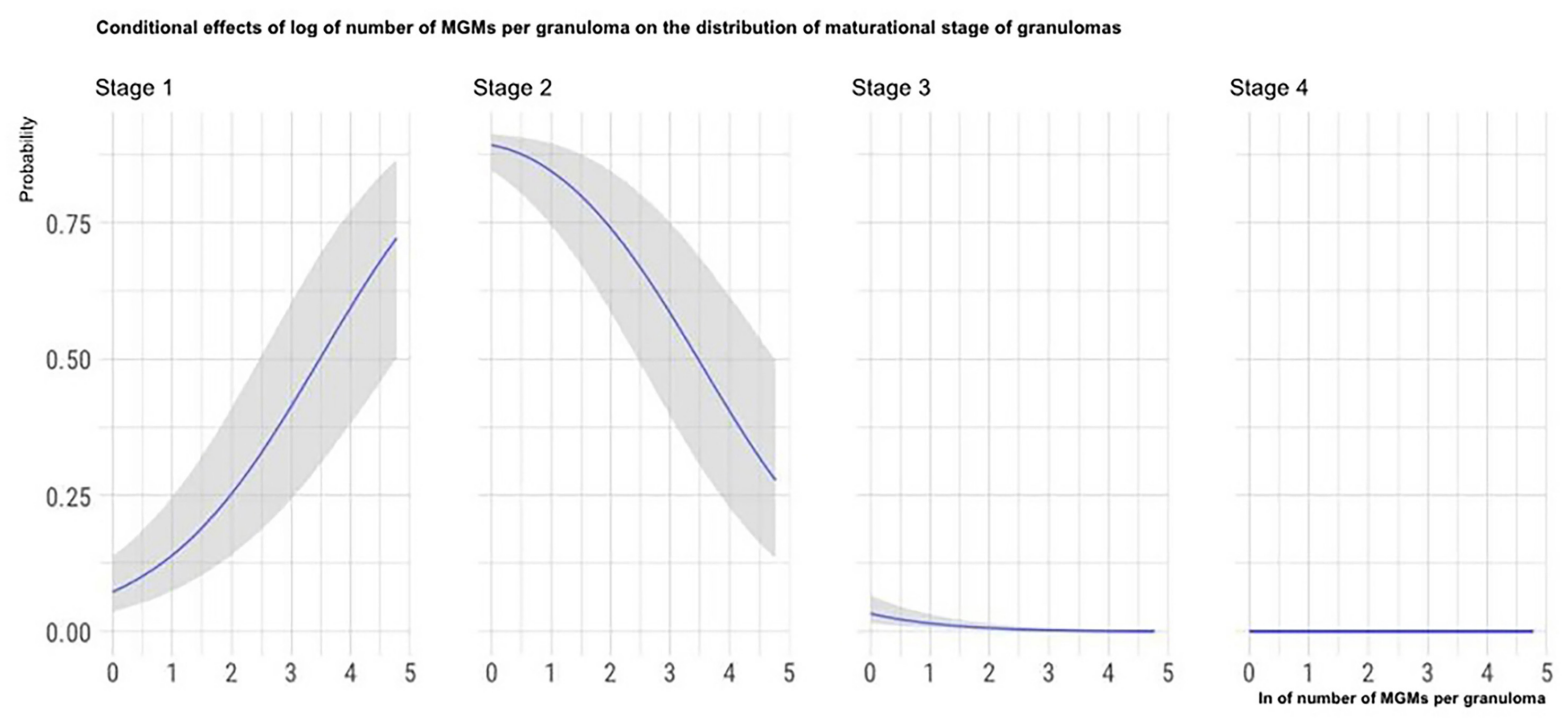
Marginal effects of the number of MGMs per granuloma on granuloma histological stage. The figure shows that as the number of MGMs per granuloma increases, the possibility that granulomas are classified in the high histological stages decreases.

The marginal effects (obtained by keeping the other predictor values constant and observing how the probability of classifying a granuloma in one of the four histological stages varies depending on the variation of the considered predictor) of granuloma size, the total number of granulomas per LN, and the number of MGMs per granuloma on the granuloma histological stage are, respectively, depicted in Figures 4–6.

## Discussion

In this study, were histologically and statistically investigated LNs of WBs naturally infected with *Mm*, a member of the MTBC whose epidemiological dynamics in WBs are still largely unknown, probably due to its difficult identification before the advent of diagnostic molecular methods (2). Specifically, in LNs in which *Mm* was demonstrated directly in fresh tissue using molecular techniques, the association between granulomas' histological features and *Mm* microbiological isolation, and between granulomas histological features and granuloma maturational stages, were investigated.

The first step was to establish the grade of the granulomas according to the scheme described by Wangoo et al. (18) and then to statistically investigate how the various maturational stages of the granulomas, as well as the presence of AF bacilli and MGMs in the LN histological section, influenced the microbiological isolation of *Mm*.

Statistical elaboration showed that, in general, as the percentage of old stage granulomas increases, the probability of a positive microbiological result decreases. Since the literature reports WB *Mm* infection is restricted to head LN with no systemic disease being described, this finding can be explained by considering the WB immune response—it seems that the granulomatous reaction in WB efficiently contains the pathogen, inactivating it over time (1, 7).

The statistical analysis also revealed that if AF bacilli are identified in an LN histological section, the considered LN is more frequently microbiologically positive for *Mm* isolation. In *Mycobacterium tuberculosis*, AF stains are reported as capable of highlighting only a small part of the whole mycobacterial population, particularly the metabolically active subpopulation (30, 31). Even if the relationship between acid-fastness and mycobacterial metabolic status has been intensely investigated only for *Mycobacterium tuberculosis*, the high genomic homology among tuberculous bacteria suggests that such observations may also be valid for the other MTBC members (32). The positive influence of AF bacteria identification on *Mm* microbiological isolation seems to confirm this hypothesis. Indeed, the microbiological isolation of *Mycobacterium tuberculosis* metabolically inactive non-AF phenotypes is reported as extremely challenging compared to the *in vitro* isolation of metabolically active AF ones (31). The observation that ZN negative histological sections are often associated with the lack of *Mm* microbiological isolation can also be explained considering that the altered cell wall of non-viable mycobacteria cannot retain fuchsin following the alcohol–acid wash (33). The two hypotheses, namely the presence of dormant non-AF mycobacteria and the presence of non-viable mycobacteria, are not mutually exclusive. Further studies are needed to understand the phenomenon thoroughly and to demonstrate the existence of metabolically inactive non-AF phenotypes in MTBC members other than *Mycobacterium tuberculosis*. Moreover, further studies (e.g., considering the number of AF bacteria and not their mere presence within the analyzed LN sections) are also necessary to better establish the association between AF bacteria and the possibility of isolating the pathogen in culture.

The presence of MGMs in the LN section negatively affects *Mm* microbiological isolation. This finding contrasts with the literature where MGMs are generally reported as a negative prognostic factor for tubercular infection containment (10, 19, 20). Indeed, MGMs act as a protective niche for the pathogen that, protected within cells unable to inactivate them, can persist viably (and therefore microbiologically isolable) within the host (34). The negative influence observed in this study can be due to the applied statistical model categorizing LNs as harboring or not harboring MGMs, independently from their number that could also be very low. Further statistical studies, considering the number of MGMs observed within LN sections, will confirm this hypothesis.

The second step of the study was to analyze histological features and microbiological results on the maturational stage of the single granulomas. In this case, the evaluated histological features were the granuloma area, the completeness of lesion within the section, the number of AF bacilli and MGMs within the single granuloma, and the total number of granulomas in the LN section.

Considering the granuloma area, the applied statistical model showed that this variable significantly influences the maturational stage of the considered granuloma: as a granuloma's area increases, the probability that the granuloma is classified in the oldest maturational stages increases. Moreover, as shown in Figure 4, the granuloma area seems to predict the maturational stage of the considered granuloma with high accuracy: the granuloma area increases with the maturational stage.

The statistic also revealed that as the granulomas stage increases, the probability that they are completely present in the histological sections decreases. This finding can be explained by considering the size of the granulomas. Indeed, during the gross sampling of LNs and afterward at the trimming of the samples for paraffin-embedding, practical issues prevent completely sampling the more extensive lesions, which, as mentioned above, usually belong to the advanced maturational stages.

Indirectly linked to the granulomas' size is the negative influence observed between the total number of granulomas in the LN sections and the granulomas' maturational stage. During the lesions' maturation, the merging of first stage granulomas can give rise to larger lesions. Again, the granulomas' fusion would also explain why in the LNs harboring few granulomas; these are more often of the higher maturational stages (18, 35).

Among the histological features analyzed in this part of the study, a negative influence of the number of MGMs on the granuloma maturational stage (i.e., a decrease of MGMs number with the increase of granulomas maturational stage) was observed. Since MGMs are phagocytic cells with a reduced bactericidal activity that are exploited by the mycobacteria as persistence and replicative niche, our results suggest that granulomas of the highest maturational stages are less permissive to the pathogen compared to the less mature ones (34). Consequently, this finding denotes that with the maturation of the granulomas, the lesions became less favorable to the pathogen survival and is consistent with the relatively high WB resistance to tuberculous infections reported in the literature (1, 7). This finding is coherent with another result of the present study. In fact, it is more frequent to isolate *Mm* microbiologically from LNs containing a higher proportion of granulomas of the first maturational stages. This result could indicate that following granuloma maturation, the number or the vitality of mycobacteria in the lesion decreases.

Concluding, in the present study LNs of *Mm* naturally infected WB were histologically and statistically evaluated. The results obtained suggest that granulomas' maturation and organization permit an efficient containment of the infection. This containment can be due to *Mm*'s characteristics, such as RD1^mic^ deletion, and those of the WB, a species known to be generally resistant to tubercular infections (1, 7, 12). Our results also suggest that the intra-species infection transmission might be an unlikely event and offer new hints that can help to explain the epidemiology of WB *Mm* infection. However, further studies considering other parameters influencing the disease transmission such as the duration of the infection, a factor potentially limiting the findings of the present study, are necessary to fully elucidate the effects of the considered parameters on the epidemiological dynamics of *Mm* infection in the WB.

## Data Availability Statement

The original contributions presented in the study are included in the article/Supplementary Material, further inquiries can be directed to the corresponding author/s.

## Ethics Statement

Ethical review and approval was not required for the animal study because it did not involve the purposeful killing of animals. Retrospective samples collected in the context of Lombardy and Emilia Romagna regions wildlife monitoring and control plan, which involves post-mortem inspection of hunted wild animals, were used. No ethical approval was deemed necessary.

## Author Contributions

CP, VT, LRG, GS, MC, and VG designed the study. CP, LRG, GS, and VG performed the histological investigation. VT performed the statistics analysis. CP, VT, LRG, and VG were involved in table and figure preparation. LRG, AG, GLA, MP, MZ, and MBB were involved in samples and data acquisition. CP, VT, and VG drafted the original paper. LRG, AG, GLA, MP, MZ, MBB, GS, MC, and VG revised and edited the manuscript. CP, GS, MC, and VG supervised and administered the project. All authors read and approved the final manuscript.

## Conflict of Interest

The authors declare that the research was conducted in the absence of any commercial or financial relationships that could be construed as a potential conflict of interest.

## Publisher's Note

All claims expressed in this article are solely those of the authors and do not necessarily represent those of their affiliated organizations, or those of the publisher, the editors and the reviewers. Any product that may be evaluated in this article, or claim that may be made by its manufacturer, is not guaranteed or endorsed by the publisher.
